# A stimuli-responsive porous carbon nanovehicle for light-initiating and thermo-driving phototheranostics of renal cell carcinoma

**DOI:** 10.1016/j.isci.2026.115701

**Published:** 2026-04-14

**Authors:** Cheng Qiu, Zhaojie Su, Mengzhen Lv, Xiyue Cao, Jianfei Xia, Runhe Zhou, Shiquan Xu

**Affiliations:** 1 Department of Organ Transplantation, Xiang’an Hospital of Xiamen University, School of Medicine, Xiamen University, Xiamen 361102, P.R. China; 2 College of Chemistry and Chemical Engineering, Shandong Sino-Japanese Center for Collaborative Research of Carbon Nanomaterials, Instrumental Analysis Center of Qingdao University, Qingdao University, Qingdao 266071, P.R. China; 3 Department of General Surgery, Qilu Hospital(Qingdao), Cheeloo College of Medicine, Shandong University, Jinan, Shandong, China

**Keywords:** chemistry, biological sciences, materials science

## Abstract

Based on DNA-gated stimuli-responsive nanoscale porous carbon, a smart phototheranostic nanovehicle, which can respond to targeted near-infrared laser irradiation, is assembled. Utilizing single-stranded DNA as a gating molecule through π-π interactions, the nanoscale porous carbon loaded with photothermal organic dyes was completely packaged. Under laser irradiation, synergistic photothermal conversion is initiated, and the temperature rises up to nearly 55°C in the designated region, which provides an ideal local condition for photothermal therapy. In the meantime, heat drives single-stranded DNA (ssDNA) detaching from the surface of NCZIF, and organic dyes are leaked for *in situ* fluorescent imaging of tumor sites. As a proof of concept, two nanovehicles, ICG@NCZIF and MB@NCZIF, are separately prepared and systematically compared. The better-performed ICG@NCZIF is further used in in vivo phototheranostics of renal cell carcinoma (RCC) and exhibits satisfactory results of tumor inhibition and accurate tumor imaging. Thus, the as-designed phototheranostics nanovehicle ICG@NCZIF holds potential for (pre)clinical translational development.

## Highlights

• A smart light-initiated thermo-driven phototheranostics nanovehicle is reported
• It uses single-stranded DNA as a gating molecule to seal organic dyes in NCZIF
• Laser irradiations generate highly efficient synergistic photothermal effects
• Targeted fluorescent imaging is achieved by thermo-driven release of dyes

## Graphical abstract

**Figure:**
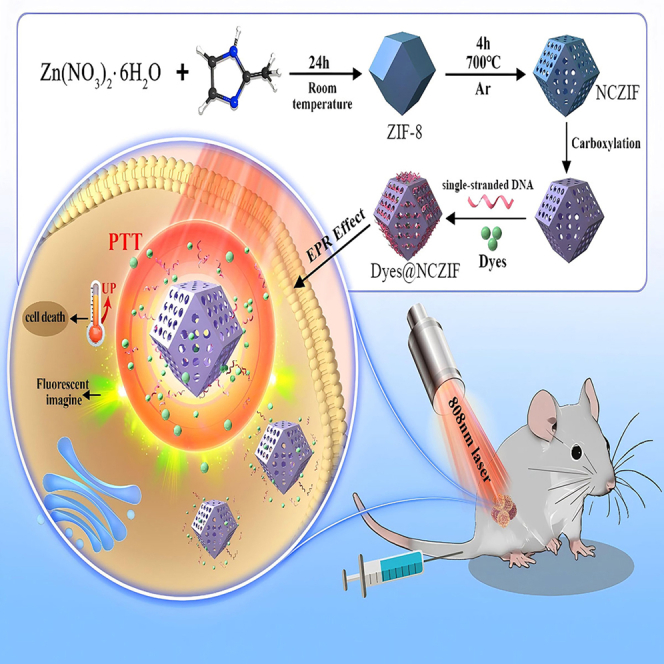

## Introduction

Renal cell carcinoma (RCC) has been widely recognized as a heterogeneous disease encompassing different subtypes and has an increasing incidence worldwide.^1^^,^^2^ Based upon the most recent cancer statistics released by the American Cancer Society (ACS), kidney cancer, including RCC, ranks the sixth highest occurring cancer among men and ninth among women. Among them, about 70–80% of RCC cases have a relatively poor prognosis, with 30% of patients developing metastatic RCC. In spite the fact that both therapy, such as immunotherapy and cell therapy, and diagnosis, such as computed tomography or magnetic resonance imaging for RCC improved dramatically in the last decade, research related to therapy and diagnosis of RCC is lagging much behind other types of cancers.

Photothermal therapy (PTT) is a type of cancer treatment by killing tumor cells via hyperthermia which originated from the conversion of light energy to heat.^3^ PTT is noninvasive, oxygen-independent compared with photodynamic therapy (PDT), and easy to operate, which was considered as excellent strategy and has many development opportunities.^4^ For RCC, especially chemotherapy/radiotherapy-resistant clear cell RCC (ccRCC), PTT has shown promising efficacy by ablating *in situ* tumors and deep-tissue lesions with minimal damage to normal renal tissue. Notably, RCC-targeted PTT is often combined with PDT, chemotherapy, or immunotherapy to enhance anti-tumor effects and inhibit recurrence, addressing the high metastasis risk of the disease.^5^^,^^6^ Briefly, implementing PTT needs photothermal agents which can convert laser irradiation into high temperature, thereby increasing the temperature of the surrounding environment.^7^ Many types of photothermal agents have been reported. Those agents roughly divided into two categories: inorganic agents and organic agents. Nanocarbon materials were the most common inorganic photothermal agents owing to its good biocompatibility, simple synthesis method, and good photothermal conversion efficiency.^8^ Besides, organic polymers,^9^^,^^10^^,^^11^ gold nanostructure,^12^ metal sulfide,^13^^,^^14^^,^^15^^,^^16^ metallic oxide nanoparticles,^17^^,^^18^^,^^19^^,^^20^^,^^21^ and organic dyes^22^^,^^23^^,^^24^ were applied in many kinds of systems. The photothermal conversion efficiency of inorganic agents should be improved compared with organic dyes. The disadvantages of organic agents, such as high photobleaching, poor photothermal stability, and high blood clearance rate, should be overcome. In order to combine the advantages of favorable biocompatibility of inorganic agents and high photothermal conversion efficiency of organic agents,^25^ we consider combining organic agents with inorganic agents.

Facile synthesis methods and low cost for synthesis nanocarbon materials should be considered. Using metal-organic frameworks (MOFs) as a template to construct a variety of nanocarbon materials with specific morphologies is a common method.^26^^,^^27^^,^^28^^,^^29^^,^^30^ Through this point, synthesized nanocarbon materials have several merits. Large specific surface area and large pore size could load more agents to improve photothermal conversion efficiency.^31^^,^^32^^,^^33^ The morphology could be adjusted via a change in experimental conditions. The removal of metal ions leaves many functionalized sites, which can be functionalized to achieve various functions.^34^ Nanoscale porous carbon derived from ZIF-8 (NCZIF) is a kind of porous nanocarbon material that has a large surface area, rich porosity, favorable biocompatibility, and the convenience of surface functionalization.^35^^,^^36^^,^^37^^,^^38^^,^^39^ The large surface area makes NCZIF load much photothermal organic agents such as indocyanine green (ICG) or methylene blue (MB) to improve photothermal conversion efficiency. In addition, NCZIFs are easy to assemble as stimuli-responsive nanovehicles with the help of biomolecule gating units of nucleic acid,^40^^,^^41^^,^^42^ which can be used for intracellular delivery and controllable release of guest molecules. All these excellent aspects of NCZIF make it a suitable carrier and photothermal agent for in situ diagnosis and PTT photothermal.

**Scheme 1:**
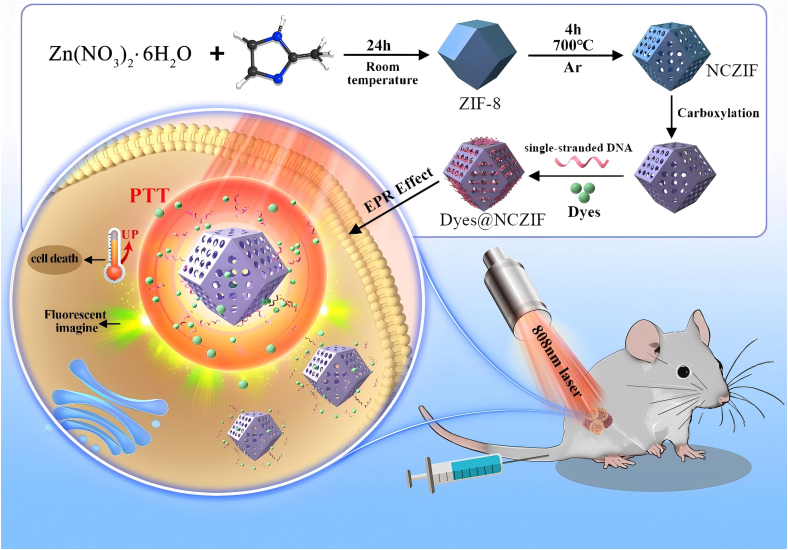
Schematic illustration of the synthesis of ICG@NCZIF (or MB@NCZIF) and its application for phototheranostics

Herein, aiming to theranostics of RCC, a simple, cost-effective, noninvasive phototheranostics vehicle composed of stimuli-responsive nanoscale porous carbon was constructed. As illustrated in Scheme 1, firstly, NCZIF was synthesized through calcining ZIF-8 and acidulation. Then, organic dyes were encapsulated in the pores of NCZIF, using single-stranded DNA (ssDNA) as a gatekeeper via π-π interaction, by means of which dyes@NCZIF nanovehicles are obtained. The targeted irradiation of the 808 nm laser plays two roles. First of all, it triggered highly efficient synergistic photothermal conversion to generate up to nearly 55°C high temperature, killing tumor cells. In the second place, the thermo-driven detaching of ssDNA from the surface of NCZIF and the release of organic dyes from the inner pores of NCZIF were induced during the PTT process, which can bring about in situ guiding fluorescent images for the PTT. As proof of concept, two commonly used dyes, ICG and MB, are loaded in NCZIF, respectively, and the PPT performance of MB@NCZIF and ICG@NCZIF was systematically examined. Finally, the ICG@NCZIF, because of better photothermal conversion efficiency, was chosen as the phototheranostics agent for *in vivo* fluorescent imaging-guided treatment of RCC.

## Results and discussion

### Characterization

**Figure 1:**
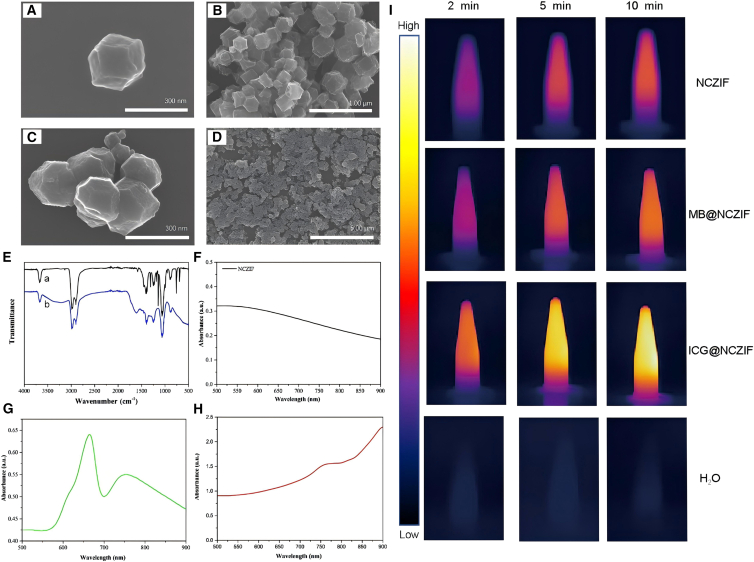
Characterization of NCZIF before and after carboxylation, as well as dyes@NCZIF. SEM images of NCZIF (A and B) and NCZIF after carboxylation (C and D). (E) FTIR spectrum of NCZIF (curve a) and NCZIF after carboxylation (curve b). Absorbance spectrum of NCZIF (F), MB@NCZIF (G) and ICG@NCZIF (H). (I) Thermal imagines of NCZIF (1 mg mL^−1^), MB@NCZIF (1 mg mL^−1^), ICG@NCZIF (1 mg mL^−1^) and H_2_O under 808 nm laser irradiation in 2.5 W cm^−2^ within 10 min.

The morphologies of nanoscale porous carbon derived from ZIF-8 and NCZIF were measured by SEM. After carboxylation (Figures 1C and 1D), the morphologies showed no conspicuous change compared to nanoscale porous carbon derived from ZIF-8 (Figures 1A and 1B). The results indicated that the procedure of carboxylation cannot destroy the morphologies of nanoscale porous carbon derived from ZIF-8, which is a dodecahedron. Figure 1E displays the FTIR images of ZIF-8 (curve a) and NCZIF (curve b). After carboxylation, the specific peak of stretching vibration of carboxyl occurred in 1609 cm^−1^, which indicated the successful fabrication of NCZIF.

The MB and ICG loading efficiency were evaluated by DLC and DLE, where DLC means molecule loading content (wt %), and DLE means molecule loading ability (wt %). DLC and DLE are calculated by following equations:

DLC=(amountofloadedmolecule/amountofmolecule−loadedNPs)×100%

DLE=(amountofloadedmolecule/amountoffeedingmolecule)×100%

Using the calibration curves of MB and ICG (Figure S3), the amount of MB and ICG in solution were measured, and then it is to be calculated that DLE_(ICG)_ = 66%, DLC_(ICG)_ = 39.8%, DLE_(MB)_ = 46% and DLC_(MB)_ = 31.5% by the above equations. These results indicated that the NCZIF has a favorable loading efficiency for further use.

### Photothermal properties

Figures 1F–1H show the absorption spectra of NCZIF, MB@NCZIF, and ICG@NCZIF, respectively. They all displayed strong NIR absorption within the range of 500 nm–900 nm. The result indicated the theoretical possibility of converting NIR light power to heat energy of the three nanomaterials mentioned above. Owing to their strong NIR absorption, NCZIF, MB@NCZIF, and ICG@NCZIF have a promising role as photothermal agents for PTT. Furthermore, in order to obtain optimal nanomaterials for combine the merits of the nanomaterials, photothermal heating curves of samples with NCZIF, MB@NCZIF, and ICG@NCZIF were determined by NIR laser irradiation (808 nm at 2.5 W cm^−2^, 10 min) and shown in Figure S4. As a result, the temperature of NCZIF increased from 26.3 °C to 47.1 °C, while MB@NCZIF only reached from 26.3 °C to 49.1 °C, which has a little increase in contrast to NCZIF. Differently, the temperature of ICG@NCZIF was increased from 26.3°C to 62°C. Water only reached from 26.3°C to 26.8°C. The results indicated that the photothermal capacity of nanomaterials we measured was determined by the type of organic dyes encapsulated by NCZIF. ICG has better photothermal ability than MB. The approximate plateau was obtained when the irradiation remained 10 min. Figure 1I showed twelve thermal photos which could directly display the temperature change of NCZIF, ICG@NCZIF, and MB@NCZIF under NIR irradiation at different times. ICG@NCZIF showed a higher temperature than both NCZIF and MB@NCZIF at 2, 5, and 10 min. At the same time, the temperature of ICG@NCZIF was increased from 2 to 10 min. These results were in keeping with the results from Figure S4. Thus, taking all of the above considerations, the laser irradiation time of 10 min was selected. As a comparison, the temperature curve of free MB and ICG were also determined (Figure S5). The temperature of free MB was increased from 26.9°C to 42.2°C, and free ICG was increased from 26.9°C to 58.4°C. They both reach a temperature plateau within 3 min. That would be due to the absence of the controlled-releasing effect of MB@NCZIF and ICG@NCZIF caused by thermo-induced uncapping of DNA from NCZIF under irradiation of a laser. Furthermore, the plateau of temperature of MB@NCZIF and ICG@NCZIF was higher than that of free MB and free ICG, which indicated that the encapsulation decreased the photo-bleaching of MB and ICG.

**Figure 2:**
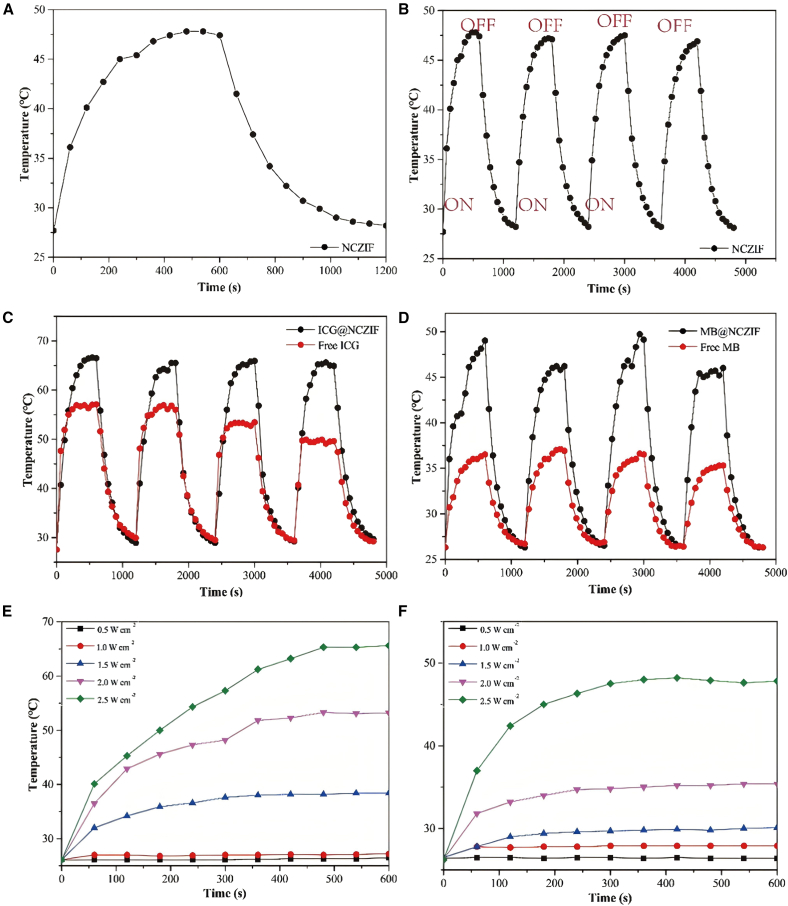
Photothermal performance of dyes@NCZIF (A) Temperature changes of NCZIF (1 mg mL^−1^) in 1 cycle. (B) Temperature changes of NCZIF (1 mg mL^−1^) in 4 cycles. (C) Temperature changes of free ICG (1 mg mL^−1^) and ICG@NCZIF (1 mg mL^−1^) in 4 cycles. (D) Temperature changes of free MB (1 mg mL^−1^) and MB@NCZIF (1 mg mL^−1^) in 4 cycles. (E) Temperature changes of ICG@NCZIF (1 mg mL^−1^) at different power densities ranging from 0.5 to 2.5 W cm^−2^. (F) Temperature changes of MB@NCZIF (1 mg mL^−1^) at different power densities ranging from 0.5 to 2.5 W cm^−2^.

The photothermal stability of NCZIF was evaluated through its periodic exposure to a laser. As shown in Figure 2A, after reaching the photothermal steady-state within 600 s, the laser power was shut down. After 4 cycles, the highest temperature each cycle was decreased by 0.9°C (Figure 2B). This indicates pure NCZIF has promising photothermal stability, which could not only help the organic dyes encapsulating in the pores of NCZIF but also has a synergistic effect owing to the stacking of heat. The photothermal stability of ICG@NCZIF and MB@NCZIF was also evaluated. As shown in Figures 2C and 2D, ICG@NCZIF shows better stability than free ICG after four cycles of laser power opening and shutting down. The highest temperature of ICG@NCZIF was decreased by 1.7 °C after four cycles, compared to free ICG, with 7.2°C of the maximum temperature difference after 4 cycles. However, the stability of MB@NCZIF was worse than that of free MB. The maximum temperature was a difference of 3.0 °C between MB@NCZIF and 0.7 °C between free MB. It may be attributed to the lower DLC. These results indicated that the photothermal stability of NCZIF successfully hindered ICG from photobleaching under laser light irradiation. Furthermore, the temperature of ICG@NCZIF and MB@NCZIF went up dramatically as the laser power density increased from 0.5 to 2.5 W cm^−2^ (Figures 2E and 2F). The results further manifested that the heat is generated by NIR irradiation of ICG@NCZIF and MB@NCZIF. The photothermal conversion efficiency (η) of ICG@NCZIF and MB@NCZIF was calculated according to previous reports.^43^ The detailed value of η was calculated. The results showed that the η of ICG@NCZIF is 76.0%, which is much higher than that of free ICG with 46.6%. This phenomenon would be attributed to the synergistic photothermal effect between encapsulated ICG and NCZIF. The time-dependent release of ICG from ICG@NCZIF was also investigated (Figure S8). The fluorescence intensity of the released ICG gradually increased over time, approaching a plateau after approximately 20 min. Meanwhile, the detectable fluorescence signal in the supernatant at 0 min can be attributed to a combination of non-specific release prior to irradiation and the initial phase of DNA dissociation triggered by the photothermal effect during the early stage of illumination.

### *In vitro* cytotoxicity and NIR PTT

**Figure 3:**
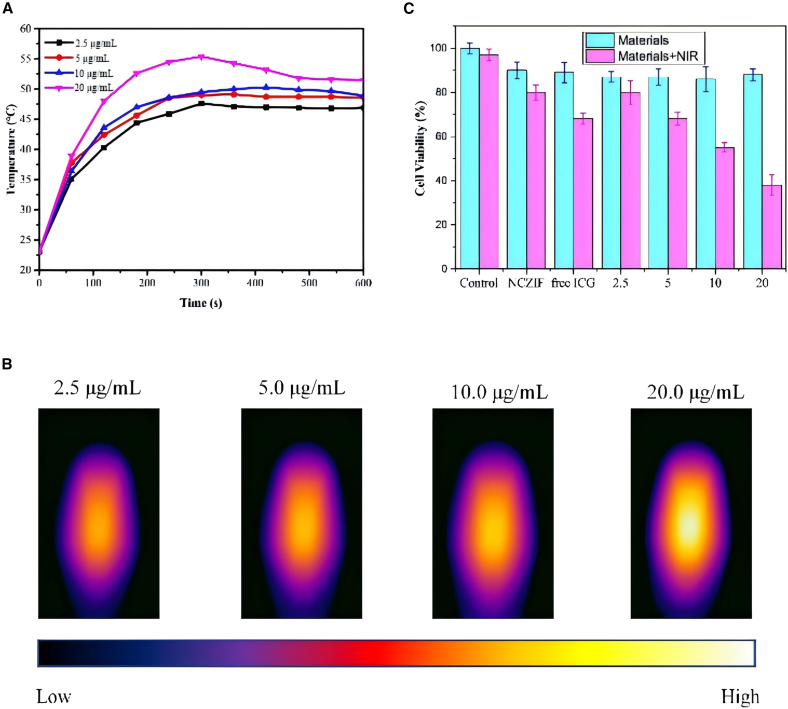
Concentration-dependent photothermal performance of ICG@NCZIF and its cytotoxic effect on HeLa cells with and without laser irradiation (A) Temperature changes of ICG@NCZIF at different concentrations ranging from 2.5 to 20 μg mL^−1^ in 10 min. (B) Thermal imagines of ICG@NCZIF at different concentrations ranging from 2.5 to 20 μg mL^−1^ at 10 min under 808 nm laser irradiation in 2.5 W cm^−2^. (C) Viability of HeLa cells incubated with NCZIF and ICG@NCZIF at different concentrations with and without 808 nm laser irradiation.

**Figure 4:**
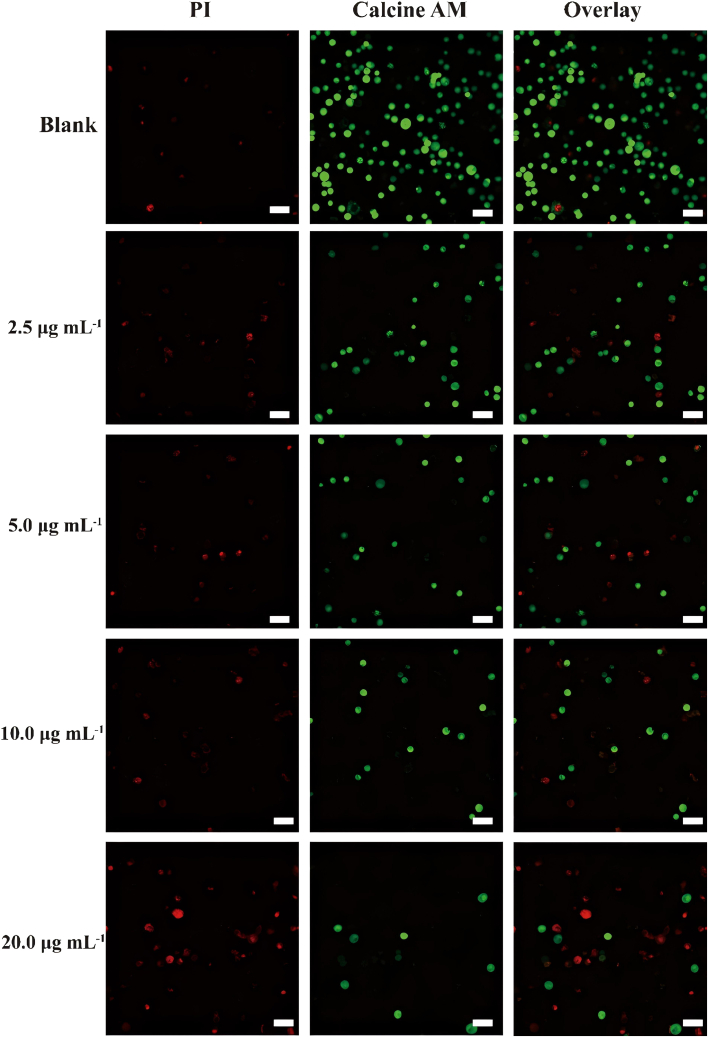
Confocal images of HeLa cells incubated with and without ICG@NCZIF with different concentrations Scale bars, 50 μm. Calcien-AM (ex/em = 490/515 nm) staining of live cells is shown in green, PI (ex/em = 535/617 nm) staining of dead cells is shown in red.

For further investigation of *in vitro* NIR PTT properties of dyes@NCZIF vehicles, ICG@NCZIF was chosen for subsequent experiments, owing to its promising photothermal capacity and brilliant stability. Firstly, for investigating the capacity of killing tumor cells of ICG@NCZIF and considering the damage of surrounding environment, avoiding high toxicity is essential. Decreasing the concentration of NCZIF and ICG while maintaining hyperthermia could help the continuation of the experiment. We chose 200 μg mL^−1^ NCZIF encapsulating ICG with different concentrations (0, 2.5, 5.0, 10.0, 20.0 μg mL^−1^) and tested the cytotoxicity. In Figure 3A, temperature change of different concentrations of ICG encapsulated in 200 μg mL^−1^ NCZIF was detected. Although decreasing the concentration of NCZIF and ICG, temperature rising to about 53°C under NIR irradiation for about 10 min can effectively create a hyperthermia environment for killing tumor cells. Figure 3B displayed thermal photos of different concentrations under 808 nm laser irradiation at 10 min. Considering the fact that the biocompatibility and PTT properties of ICG@NCZIF were appropriate, *in vitro* photothermal property against Hella cells was assessed using CCK-8 assay. In Figure 3C, the blue column shows the cytotoxicity of materials. It can be seen that NCZIF (200 μg mL^−1^), free ICG (20 μg mL^−1^), and ICG@NCZIF with different concentrations of ICG all display remarkable biocompatibility without NIR irradiation. Figure 3C also showed the PTT effects of NCZIF, ICG, and ICG@NCZIF at different concentrations. The concentration of NCZIF is 200 μg mL^−1^ and the concentration of free ICG is 20 μg mL^−1^. Only NCZIF exists, 200 μg mL^−1^ NCZIF under 808 nm laser irradiation for 10 min could kill only about 20% HeLa cells. Free ICG with 20 μg mL^−1^ could kill about 32% HeLa cells. Owing to the synergistic effect, ICG@NCZIF displayed greater advantage in eliminating cancer cells with a brilliant decrease in cell viability up to 38% at ICG concentrations of 20 μg mL^−1^. To better visualize the feasibility of cell inhibition with ICG@NCZIF, a confocal laser scanning microscope was used to take the fluorescent photos after different treatments of ICG@NCZIF. HeLa cells were incubated with different groups, and subsequently co-stained with calcein-AM and PI to differentiate live cells (green) and dead cells (red). As shown in Figure 4, strong green fluorescence with no treatment in the blank group was displayed, showing that a good situation of HeLa the blank group was displayed, showing a good situation of HeLa cells in the medium. However, cells with ICG@NCZIF under laser irradiation suggested partial cell death. At a concentration of 2.5 μg mL^−1^, approximately 15% of cells were killed, while increasing the concentration to 20 μg mL^−1^ resulted in about 90% cell death. These results clearly demonstrate that when the concentration of ICG was increased, dead cells increased, indicating its promising potential for cancer therapy.

### *In vivo* NIR phototheranostics and biocompatibility

**Figure 5:**
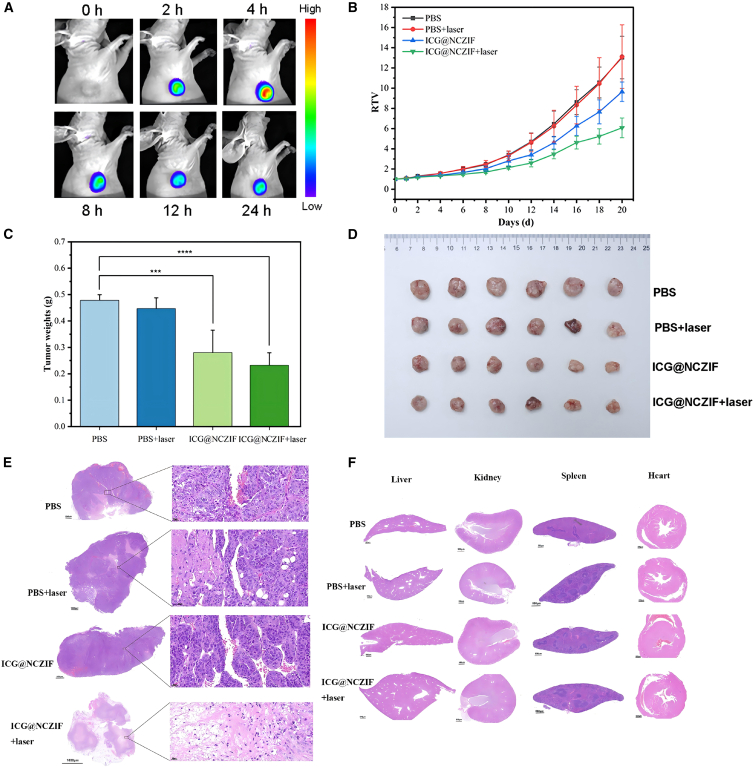
Fluorescence imaging, photothermal therapy and histological assessment *in vivo* (A) fluorescent images of tumors tissue (arrows) at different time points (0, 2, 4, 8, 12 and 24 h) after injection of ICG@NCZIF via tail vein under 745 nm laser irradiation. (B) Tumor volume growth curves of every group of mice after NIR laser therapy. (C) Average weights of tumors obtained from the mice at the end of photothermal therapy. (D) Representative photos of mice at the end of treatment. (E) Pictures of H&E-stained tumors from mice in the four groups. (F) Pictures of H&E-stained organs (liver, kidney, spleen and heart) from mice in the four groups.

With the photothermal process of ICG@NCZIF, the thermo-induced DNA stripping from the surface of ICG@NCZIF occurred, by means of which, the encapsulated ICG partly leaked from ICG@NCZIF, which can generate a fluorescent signal for in situ imaging, endowing PTT ICG@NCZIF with phototheranostics ability. As shown in Figure 5A, *in vivo* fluorescent imaging of renal tumor sites can be clearly observed after ICG@NCZIF is injected in modeled mice after 2 h. In addition, the metabolism of ICG@NCZIF inside the tumor was also supervised. Due to the low fluorescence emission at the excitation wavelength of 745 nm, a barely fluorescent signal was detected in the tumor region before injection. After ICG@NCZIF injection, the fluorescent signal at the tumor site was significantly enhanced from 2 to 4 h post-injection, and then it declined from 4 to 24 h because of the metabolism of ICG@NCZIF. At 4 h post-injection, the fluorescent signal reached a maximum value. These results indicated that the ICG@NCZIF performs significant passive targeting and has a long residence time in renal tumors, which would contribute to efficient enhanced permeability and retention (EPR) effect caused by surface and appropriate particle size of ICG@NCZIF, the protection from ICG blenching, and controlled-release of ICG. The PTT of ICG@NCZIF *in vivo* was further studied. ACHN tumor-bearing mice were divided into four groups randomly. (1) PBS (i.v. injection), (2) PBS (i.v. injection)+808 nm laser, (3) ICG@NCZIF (i.v. injection), and (iv) ICG@NCZIF (i.v. injection) +808 nm laser. The tumor volumes of different groups were measured and recorded every other day during the subsequent 20 days (Figure 5B). On the 10th day, group (4) started to show efficient tumor restraint and maintained a pretty low relative tumor volume (RTV) increasing rate. Under comparisons, group (1), (2), and (3) showed relatively rapid growth rates, indicating that neither ICG@NCZIF nor laser significantly restrained the tumor development. After 20 days PTT, the tumor weights and tumor photographs in each group were shown in Figures 5C and 5D. The tumor volume and weight in group (4) were the lowest values among all the groups, further demonstrating the effectiveness of the treatment. The tumor tissues were studied through a pathological examination by hematoxylin and eosin (H&E) staining (Figure 5E). Compared with other control groups, the group (4) showed higher cell necrosis, including pyknosis, karyorrhexis, and karyolysis. Above all, ICG@NCZIF had good performance in fluorescent imaging, and PTT inhibition of renal tumor metastasis, which indicates its great potential for use as a powerful tool for phototheranostics of RCC.

*In vivo* toxicity is a critical factor for the phototheranostics agent. In order to evaluate the *in vivo* toxicity of ICG@NCZIF, the pathological examination of major organs of mice by H&E staining after treatment was carried out. The liver, kidney, spleen, and heart had no obvious organ damage or tissue denaturation with ICG@NCZIF (Figure 5F). Furthermore, there were no significant difference compared to the control group in the function markers of liver (ALT and AST) and kidney (UA and BUN) (Figures S6 and S7), suggesting that no noticeable hepatic or renal dysfunction was induced by ICG@NCZIF treatments. Altogether, ICG@NCZIF showed favorable biocompatibility and can have acted as phototheranostics agents in fluorescent imaging-guided PTT of RCC.

### Limitations of the study

In summary, a novel smart nanovehicle for fluorescent imaging-guided PTT therapy is successfully fabricated. Combining two kinds of photothermal agents, the as-prepared both MB@NCZIF and ICG@NCZIF have remarkably enhanced photothermal efficiency and anti-photobleaching ability, because of the synergistic photothermal effect and protection by NCZIF. Under the near-infrared irradiation of a laser, PTT therapy and the thermo-driven fluorescent imaging-guiding process can be initiated, during which the nanovehicle ICG@NCZIF has demonstrated remarkable efficacy in eradicating renal carcinoma cells and inhibiting tumor growth, while also providing highly spatially resolved fluorescent images of tumor locations. We believe the report of a novel light-initiated thermo-driven nanovehicle, ICG@NCZIF, has yielded a valuable solution for phototheranostics of RCC.

Despite demonstrating that MB@NCZIF and ICG@NCZIF possess significantly enhanced photothermal efficiency and anti-photobleaching ability, with ICG@NCZIF showing remarkable efficacy in eradicating renal carcinoma cells and inhibiting tumor growth while enabling high-resolution fluorescence imaging, several limitations should be acknowledged. First, the tumor accumulation of ICG@NCZIF primarily relies on the passive EPR effect, and the *in vivo* evaluation was conducted using a simple subcutaneous tumor model rather than more clinically relevant orthotopic models. Additionally, long-term potential chronic toxicity of the porous carbon-based nanocarrier was not thoroughly investigated. In future work, we aim to address these limitations by constructing actively targeted NCZIF-based nanovehicles and evaluating their phototheranostic performance in orthotopic tumor models, with a focus on long-term safety under more physiologically relevant conditions.

## Resource availability

### Lead contact

Lead contact Shiquan Xu (15980913167@163.com).

### Materials availability

The materials reported in this work were synthesized in our laboratory as detailed in the experimental section.

### Data and code availability

• All data generated or analyzed during this study are included in this published article and its supplemental information files.
• This paper does not report original code.
• This study did not generate new unique reagents, materials, or other resources. All materials used are described in detail in the “STAR Methods” section.

## Acknowledgments

The authors really appreciate the financial support by the 10.13039/501100017686Fujian Provincial Health Technology project (2024QNB019), the 10.13039/501100019081Natural Science and Technology Major Project of the Xiamen (no. 3502Z20231034), and the 10.13039/100014717National Natural Science Foundation of China (82500801).

The animal study was reviewed and approved by The Institutional Animal Care and Use Committee of Xiamen University (XMULAC20190113).

## Author contributions

Conceptualization, methodology, investigation and writing – original draft, C.Q., Z.S., M.L., R.Z., and S.X.; supervision, R.Z., and S.X.; Writing—review and editing, X.C. and J.X.

## Declaration of interests

The authors state no conflict of interest.

## STAR★Methods

### Key resources table

**Table:** 

REAGENT or RESOURCE	SOURCE	IDENTIFIER
**Chemicals, peptides, and recombinant proteins**		

Zn(NO_3_)_2_.6H_2_0	Aladdin Reagent Co., Ltd. (China)	10196-18-6
2-Methyl-Imidazol	Macklin Biochemical Co., Ltd. (China)	693-98-1
Indocyanine green (ICG)	Sangon Biotech Co., Ltd. (China)	3599-32-4
Methylene blue (MB)	Sangon Biotech Co., Ltd. (China)	7220-79-3
DMEM medium	Wuhan Pricella Biotechnology Co., Ltd.	PM150210
Penicillin-Streptomycin liquid	New Cell & Molecular Biotech Co., Ltd. (China)	C100C5
Fetal Bovine Serum	Wuhan Pricella Biotechnology Co., Ltd.	164210–50
Phosphate-buffered saline	Beijing LanJieke Technology Co., Ltd.	BL302A

**Experimental models: Cell lines**		

HeLa cells	Haixing Biotech Co., Ltd. (China)	wt/wt
ACHN cells	Haixing Biotech Co., Ltd. (China)	wt/wt

**Experimental models: Organisms/strains**		

Male BALB/c nude mice	SPF Biotechnology Co., Ltd.	wt/wt

**Oligonucleotides**		

5′-CTCCTGTAATGAAGCGCTAAGTGTAATGG-3′	Sangon Biotech Co., Ltd. (China)	113324179

**Other**		

Scanning electron microscope	JEOL Co., Ltd. (Japan)	JSM-6390LV
FT-IR spectroscopy	Thermo Co., Ltd. (America)	NICOLET iS50
UV-vis spectrophotometer	Shimadzu Co., Ltd. (Japan)	UV-2550
X-ray Diffractometer	Rigaku Co., Ltd. (Japan)	SmartLab
Microplate Reader	Molecular Devices Co., Ltd. (America)	SpectraMax i3x
Laser Confocal Microscope	ZEISS Co., Ltd. (Germany)	LSM 900&Axio Imager M2

### Experimental model and study participant details

#### Cell culture

The HeLa and ACHN cells were grown in a DMEM medium (Wuhan Pricella Biotechnology Co., Ltd., China) and supplemented with 10% FBS (Wuhan Pricella Biotechnology Co., Ltd., China) and 1% penicillin - streptomycin liquid (New Cell & Molecular Biotech, China). They were cultured in an incubator (Thermo Fisher Scientific Co., Ltd., USA) at 37°C with 5% CO_2_.

#### Animal experiments

Male BALB/c nude mice (6–8 weeks) were purchased from SPF Biotechnology Co., Ltd. and used under the protocols approved by the institutional ethical committee of Xiang’an Hospital of Xiamen University (XMULAC20190113). To develop a tumor model, ACHN cells (2×10^6^ cells suspended in 200 μL of PBS) were injected subcutaneously into the fat pads of the right lower limb extremity of each mouse.

### Method details

#### Preparation of NCZIF

The 20 mL Zn(NO_3_)_2_·6H_2_O (1.49 g) aqueous solution was well mixed with the 180 mL 2-methylimidazole (24.60 g) aqueous solution. And the mixture was stirred for 24 h at 25°C. After that, the obtained crystal was washed three times with a mixture of ethanol and water at a ratio of 1:1, and the resulting white ZIF-8 powder was vacuum dried for 12 h at 60°C. A one-step method was utilized to place 300 mg of ZIF-8 nanomaterials in a tubular temperature-programmed furnace directly. Then carbonized at 700°C in an Ar environment. After 4 h, it was cooled to 25°C in Ar atmosphere to obtain black powder NCZIF. NCZIF (70 mg) was dissolved in 30 mL aqueous solution containing (NH_4_)_2_S_2_O_8_ (6.85 g) and concentrated H_2_SO_4_ (3.5 mL), then stirred at 25°C for 4 h. After washing with secondary distilled water and ethanol, drying the materials, and the brownish black carboxylated NCZIF was obtained.

#### Preparation of ICG@NCZIF and MB@NCZIF

Solution A: 1 mg NCZIF was added in 1 mL PBS (0.01 M) solution. Solution B: 1 mg ICG was added in 1 mL secondary distilled water. Solution C: 1 mg MB was added in 1 mL secondary distilled water. Put B into A and 10 μL ssDNA was added. After incubated for 24 h, the mixed compound was centrifuged and washed three times. The obtained ICG@NCZIF (1 mg mL^−1^) was dispersed in 1 mL secondary distilled water. The MB@NCZIF (1 mg mL^−1^) was constructed using the same way as ICG@NCZIF. To determine the loading efficiency of MB and ICG, UV absorbance of MB@NCZIF and ICG@NCZIF were measured in secondary distilled water at 664 nm and 775 nm, respectively.

#### NIR triggered photothermal effect

NCZIF (1 mg mL^−1^), free ICG (1 mg mL^−1^), free MB (1 mg mL^−1^), ICG@NCZIF (1 mg mL^−1^) and MB@NCZIF (1 mg mL^−1^) were put into conical centrifuge tube. Then, the 808 nm NIR laser (2.5 W cm^−2^) light irradiated the materials for 10 min. The temperature of the solutions was recorded by thermal imager (FOTRIC 225s) every 60 s. The secondary distilled water was used as control. For measuring the photothermal stability of ICG@NCZIF (1 mg mL^−1^) and MB@NCZIF (1 mg mL^−1^), free ICG (1 mg mL^−1^), free MB (1 mg mL^−1^), NCZIF (1 mg mL^−1^), ICG@NCZIF (1 mg mL^−1^) and MB@NCZIF (1 mg mL^−1^) were irradiated with 808 nm laser light for 10 min, followed by naturally falling into room temperature without laser irradiation in 10 min as one cycle. The temperature of the above solutions was recorded by thermal imager every 60 s and lasted 4 cycles. The ICG@NCZIF and MB@NCZIF were also irradiated by 808 nm laser with different laser power densities (0.5, 1.0, 1.5, 2.0, 2.5 W cm^−2^). The temperature of the solution was measured every 60 s with the thermal imager.

#### Calculation of photothermal conversion efficiency

The system has total energy balance:

(Equation 1) $$\sum_{i}m_{i}C_{p,i}\frac{dT}{dt}=Q_{NPs}+Q_{s}-Q_{loss}$$

Herein, m represents the mass, C_p_ is heat capacity of solvent (water). T is the solvent temperature. Q_np_ is the photothermal energy input by ICG@NCZIF and MB@NCZIF.

(Equation 2) $$Q_{NPs}=I\left(1-10^{-A_{\lambda }}\right)\eta$$

I is laser power, A_λ_ is the absorbance of ICG@NCZIF and MB@NCZIF with the wavelength of 808 nm. η is the conversion efficiency from the absorbed light energy to thermal energy. Q_loss_ is thermal energy lost to the surroundings:

(Equation 3) $$Q_{loss}=\text{hA}\Delta T$$

Where h is the heat transfer coefficient, A is the surface area of the container, and ΔT is the changed temperature which is referred to T-T_surr_ (T is the solvent temperature and T_surr_ is the ambient temperature of the surrounding).

Qs is the heat which associated with the light absorbed by solvent per second. the heat input is equal to the heat output at the maximum steady-statue temperature in the situation of heating pure water. Then, we got the equation as follows:

(Equation 4) $$Q_{s}=Q_{loss}=\text{hA}\Delta T_{\max,H_{2}O}$$

$\Delta T_{\max,H_{2}O}$ is the temperature change of water at the maximum steady-state temperature.

The heat inputs are the heat generated by nanoparticles (Q_NPs_) and the heat generated by water (Q_s_), which is equal to the heat output at the maximum steady-statue temperature, so the equation can be:

(Equation 5) $$Q_{NPs}+Q_{s}=Q_{loss}=\text{hA}\Delta T$$

Where ΔT_*max*,*mix*_ is the temperature change of ICG@NCZIF and MB@NCZIF solvent as the maximum steady-state temperature. According to the (Equation 2), (Equation 4), (Equation 5), the photothermal conversion efficiency (η) can be expressed as following:

(Equation 6) $$\eta =\frac{hA\Delta T_{\max,mix}-\text{hA}\Delta T_{\max,H_{2}O}}{I\left(1-10^{-A_{\lambda }}\right)}=\frac{\text{hA}\left(\Delta T_{\max,mix}-\Delta T_{\max,H_{2}O}\right)}{I\left(1-10^{-A_{\lambda }}\right)}$$

We can see that in the equation above, only hA is unknown. In order to get hA, we introduce *θ* which is defined as the ratio of ΔT to ΔT_*mix*_:

(Equation 7) $$\theta =\frac{\Delta T}{\Delta T_{mix}}$$

Substituting Equation 10 into Equation 4:

(Equation 8) $$\frac{d_{\theta }}{d_{t}}=\frac{hA}{\sum_{i}m_{i}C_{p,i}}\left[\frac{Q_{NPs}+Q_{s}}{hA\Delta T_{\max}}-\theta \right]$$

When the laser was shut off, the *Q*_*NPs*_+*Q*_*s*_ = 0, the Equation 8 can be shown as:

(Equation 9) $$d_{t}=-\frac{\sum_{i}m_{i}C_{p,i}}{hA}\frac{d_{\theta }}{\theta }$$

Equation 9 changes the expression:

(Equation 10) $$t=-\frac{\sum_{i}m_{i}C_{p,i}}{hA}\ln\;\theta$$

Where $\frac{\sum_{i}m_{i}C_{p,i}}{hA}$ can be calculated by linear relationship of time versus -*lnθ*. Then, substituting the value of *hA* into the Equation 6, the photothermal efficiency (η) of ICG@NCZIF and MB@NCZIF could be calculated. According to the equation above, η value of ICG@NCZIF was calculated to be about 76.0% and the MB@NCZIF was calculated to be about 75.9%. Then, the photothermal efficiency of free MB and free ICG were also calculated. Using the same method, η value of free ICG and free MB were both calculated. The value was about 46.6% of free ICG and about 28.5% of free MB.

#### Cytotoxicity study *in vitro*

For the *in vitro* cytotoxicity assay, HeLa cells were seeded in 96-well plates at a density of 1×10^4^ per well. Then, cells were cultured in 100 μL of DMEM medium containing 10% FBS and incubated at 37°C with 5% CO_2_ for 24 h. After removing the medium, cells were treated with DMEM medium without 10% FBS containing ICG@NCZIF at different concentrations ranges from 0 to 20 μg mL^−1^ for 4 h. All cell viabilities were assessed by Cell Counting Kit (CCK-8) assay.

#### NIR photothermal therapy *in vitro*

HeLa cells were seeded in 96-well plates at a density of 1×10^4^ per well. Then, cells were cultured in 100 μL of DMEM medium containing 10% FBS and incubated at 37°C with 5% CO_2_ for 24 h. After removing the medium, cells were treated with DMEM medium without FBS containing ICG@NCZIF at different concentrations ranges from 0 to 20 μg mL^−1^ for 4 h. Then, the cells were exposed to 808 nm laser irradiation (2.5 W cm^−2^, 10min). Cell viabilities were assessed by CCK-8 assay. Cell viability was determined using the following equation:

$$\text{Cell}\;\text{viability}\;\left(\%\right)=\frac{Absorbance_{sample}}{Absorbance_{control}}\times 100\%$$

The photothermal therapeutic effect of ICG@NCZIF was evaluated with confocal laser scanning microscope. HeLa cells were respectively treated in five groups named Blank and ICG@NCZIF with different concentrations. The cells were seeded into 96-well plates. The culture medium containing samples was respectively added to the corresponding groups and incubated for 2h. Subsequently, the culture medium was replaced. The samples from Blank and ICG@NCZIF with different concentrations were further irradiated with 808 nm laser (2.5 W cm^−2^) for 10 min. Then, cells were incubated for another 1h. At last, cells were stained with calcein-AM solution (10 μM) and propidium iodide (PI) solution (10 μM) for 15 min to display living cells (green) and dead cells (red), respectively.

#### NIR fluorescent imaging and photothermal therapy *in vivo*

ICG@NCZIF (200 μL, 50 μg mL^−1^) were injected i.v. into the molded mice. The fluorescent images were obtained and analyzed as a function of injection times (0, 2, 4, 8, 12 and 24 h) with an excitation wavelength of 745 nm after 808 nm laser irradiation on the tumor sites for 10 min. The molded mice were randomly divided into four groups (*n* = 6 per group) and treated, respectively, with (1) PBS, (2) PBS+808 nm laser irradiation, (3) ICG@NCZIF, (4) ICG@NCZIF+808 nm laser irradiation. In group (2) and (4), after the intravenous injection of PBS or ICG@NCZIF, the tumor sites were irradiated by a laser (808 nm) for 10 min as the therapeutic process (0–20 days). After the treatment, the tumor volumes and mouse body weights were measured every 2 days.

### Quantification and statistical analysis

All graphs presented in the main text and supplemental information were generated from raw data using OriginPro 2018.
