# Supplementary material for: A stimuli-responsive porous carbon nanovehicle for light-initiating and thermo-driving phototheranostics of renal cell carcinoma
Source: iScience. 2026 Apr 14;29(5):115701. doi: 10.1016/j.isci.2026.115701 (PMC13146533; doi:10.1016/j.isci.2026.115701)
Supplement: Document S1. Figures S1–S8 [file mmc1.pdf]

iScience, Volume *29*

Supplemental information

A stimuli-responsive porous carbon nanovehicle for light-initiating and thermo-driving phototheranostics of renal cell carcinoma

Cheng Qiu, Zhaojie Su, Mengzhen Lv, Xiyue Cao, Jianfei Xia, Runhe Zhou, and Shiquan Xu

Supporting Information

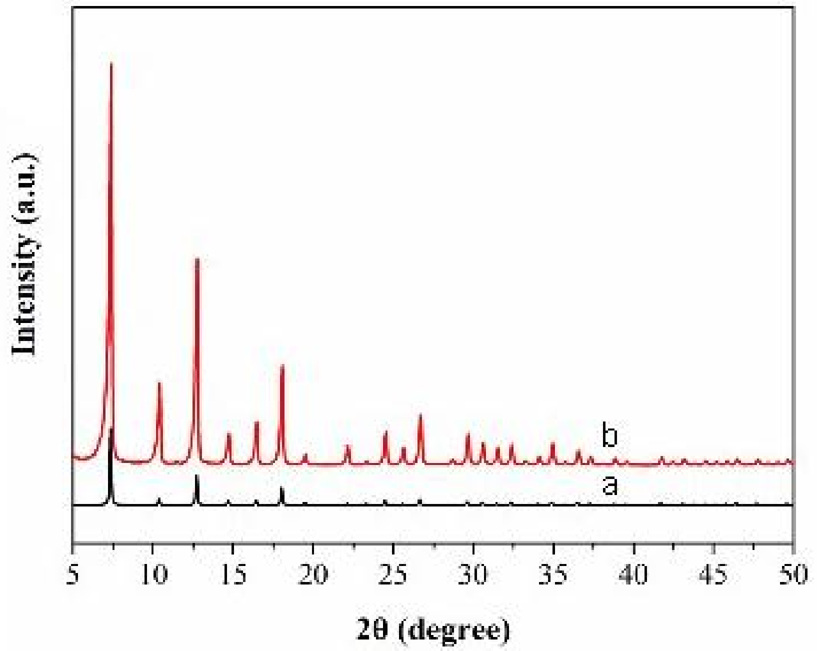

Figure S1 PXRD patterns of (a) simulated and (b) synthesized ZIF-8.

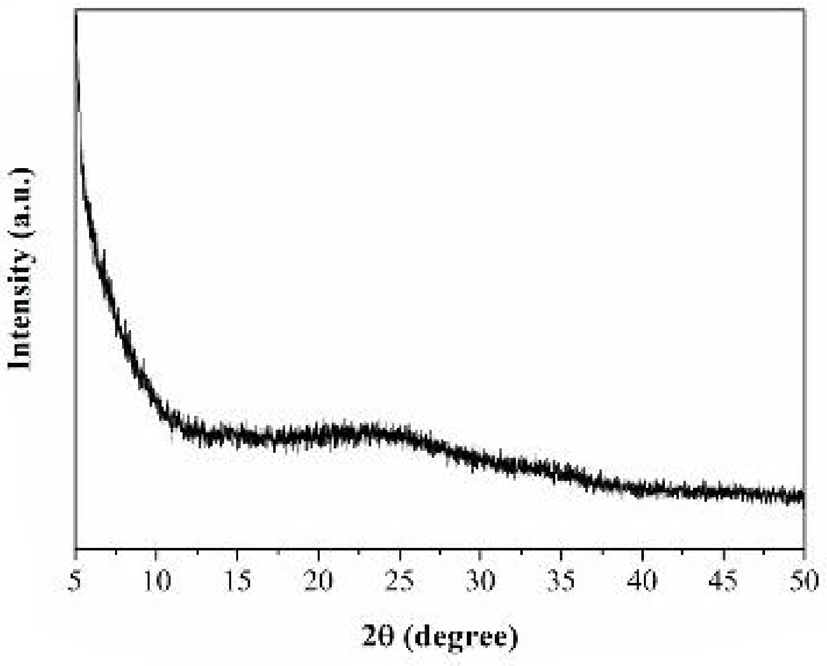

Figure S2 PXRD of NCZI

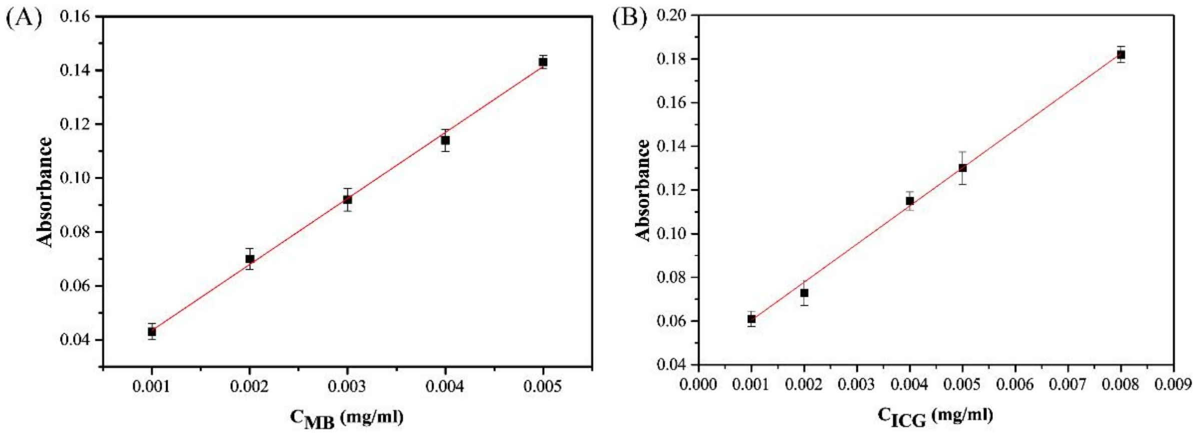

Figure S3 calibration curves of (A) MB, (B) ICG.

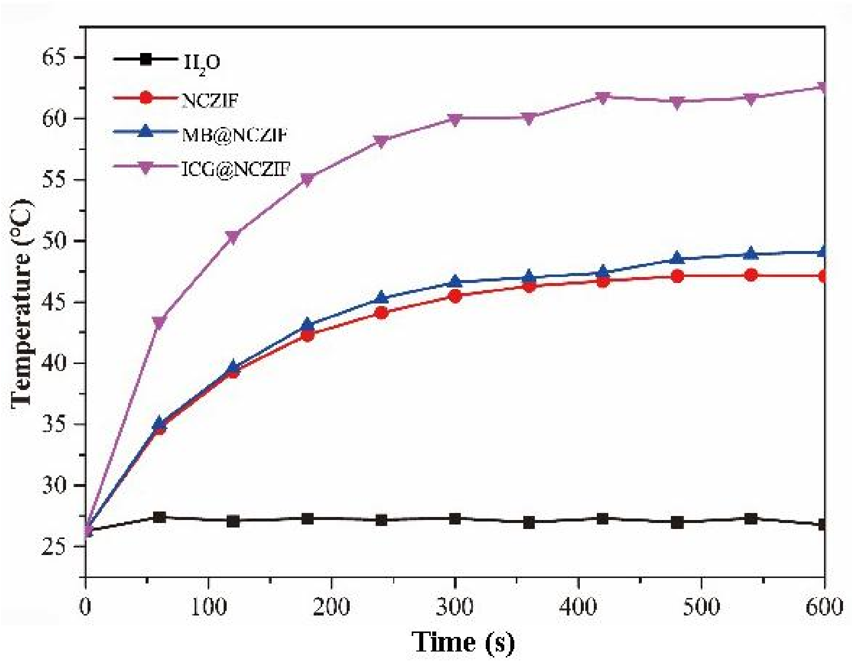

Figure S4 Temperature changes of H_2O, NCZIF (1 mg mL^{-1}), MB@NCZIF (1 mg mL^{-1}) and ICG@NCZIF (1 mg mL^{-1}) in 10 min.

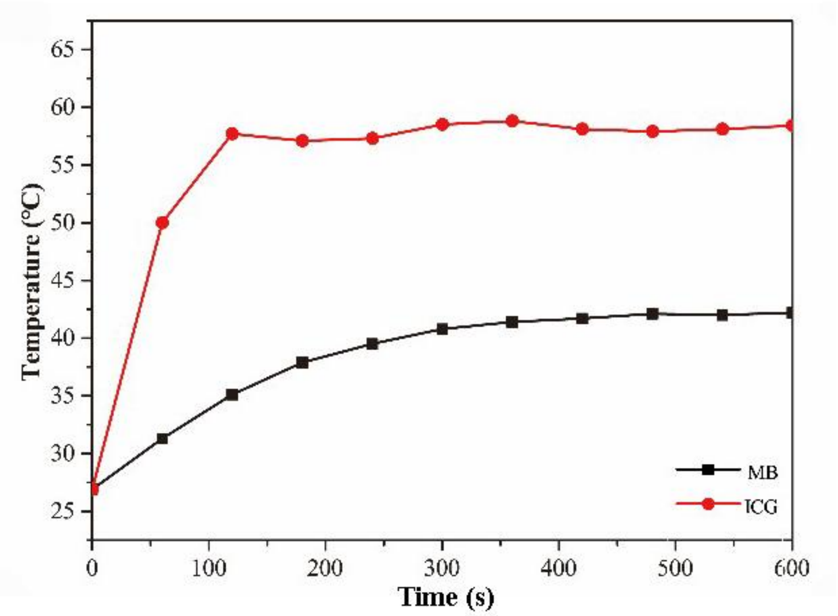

Figure S5 Temperature changes of free MB (1 mg mL^{-1}) and free ICG (1 mg mL^{-1}) in 10 min.

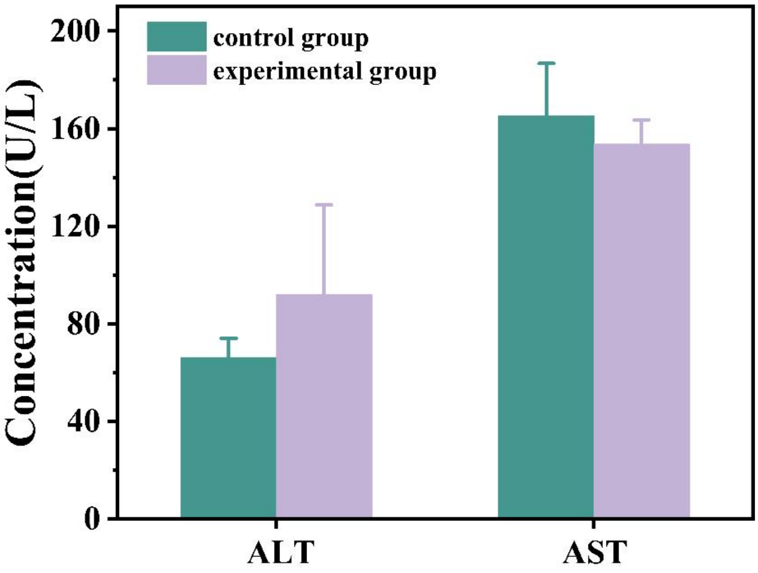

Figure S6 Liver Function markers of ALT and AST of the mice treated with ICG@NCZIF.

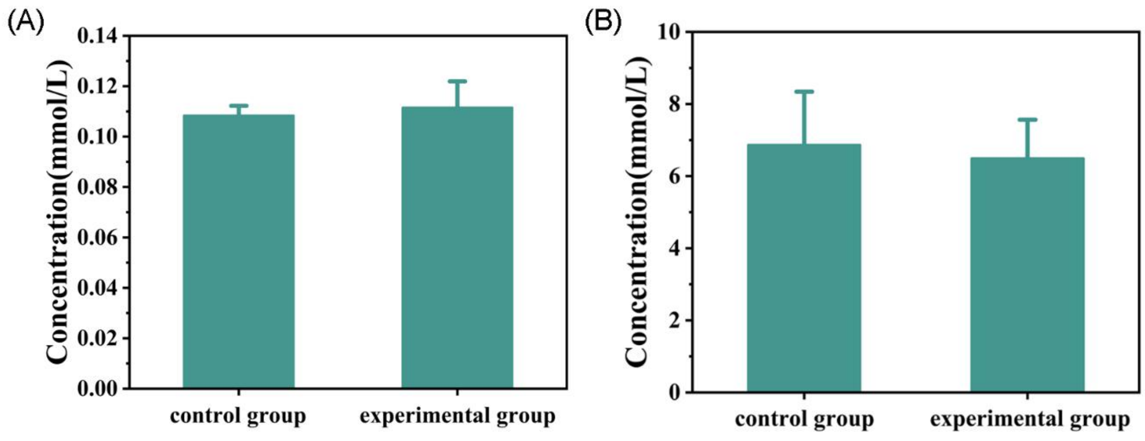

Figure S7 Kidney Function markers of (A) UA and (B) BUN of the mice treated with ICG@NCZIF.

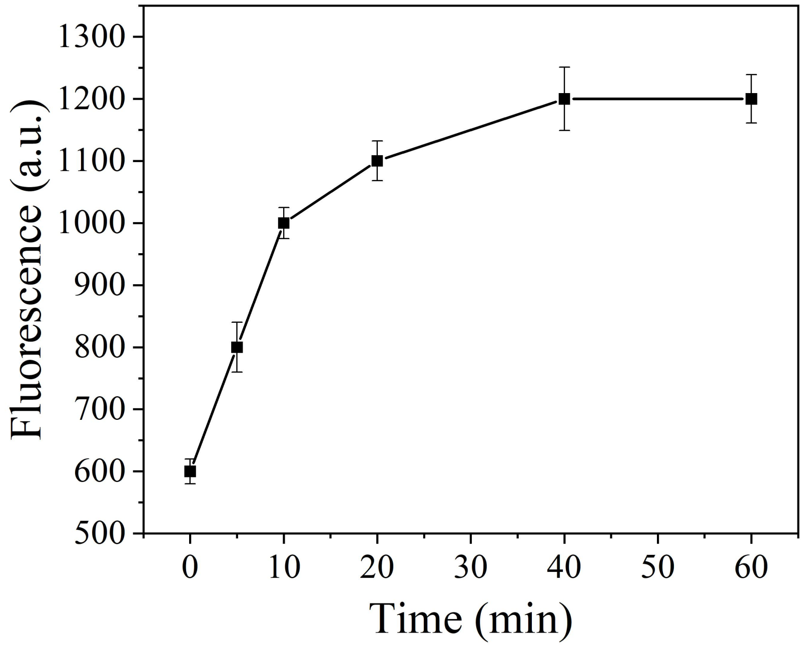

Figure S8 Time-dependent releasing of ICG@NCZIF after laser irradiation
